# *IKZF1* and *BTG1* silencing reduces glucocorticoid response in B-cell precursor acute leukemia cell line

**DOI:** 10.1016/j.htct.2024.05.004

**Published:** 2024-07-26

**Authors:** Amanda de Albuquerque, Bruno A. Lopes, Renan Amphilophio Fernandes, Etel Rodrigues Pereira Gimba, Mariana Emerenciano

**Affiliations:** a Division of Clinical Research and Technological Development, Instituto Nacional de Câncer (INCA), Rio de Janeiro, RJ, Brazil; b Genetics of Acute Leukemia Laboratory, Molecular Carcinogenesis Program, Instituto Nacional de Câncer (INCA), Rio de Janeiro, RJ, Brazil; c Pharmacology and Medicinal Chemistry Program, Institute of Biological Sciences, Universidade Federal do Rio de Janeiro (UFRJ), Rio de Janeiro, RJ, Brazil; d Department of Natural Sciences (RCN), Institute of Humanities and Health (IHS), Universidade Federal Fluminense (UFF), Rio de Janeiro, Brazil; e Hematology-Molecular Oncology Program, Research Coordination, Instituto Nacional de Câncer (INCA), Rio de Janeiro, Brazil

**Keywords:** Acute lymphoblastic leukemia, *IKZF1*, *BTG1*, Glucocorticoid, Dexamethasone

## Abstract

**Introduction:**

Secondary genetic alterations, which contribute to the dysregulation of cell cycle progression and lymphoid specialization, are frequently observed in B-cell precursor acute lymphoblastic leukemia (B-ALL). As *IKZF1* and *BTG1* deletions are associated with a worse outcome in B-ALL, this study aimed to address whether they synergistically promote glucocorticoid resistance.

**Methods:**

Small interfering RNA was used to downregulate either *IKZF1*, or *BTG1*, or both genes in the 207 B-ALL cell line. Cell viability was investigated by 3-[4,5-dimethylthiazol-2-yl]-2,5-diphenyltetrazolium bromide (MTT) and trypan blue exclusion assays. The expression levels of *IKZF1, BTG1* and glucocorticoid-responsive genes (*DUSP1, SGK1, FBXW7* and *NR3C1*) were evaluated by real time quantitative real time polymerase chain reaction (PCR).

**Results:**

Isolated silencing of *BTG1, IKZF1*, or both genes in combination under dexamethasone treatment increased cell viability by 24%, 40% and 84%, respectively. Although *BTG1* silencing did not alter the expression of glucocorticoid-responsive genes, *IKZF1* knockdown decreased the transcript levels of *DUSP1* (2.6-fold), *SGK1* (1.8-fold), *FBXW7* (2.2-fold) and *NR3C1* (1.7-fold). The expression of glucocorticoid-responsive genes reached even lower levels (reducing 2.4-4 fold) when *IKZF1* and *BTG1* silencing occurred in combination.

**Conclusions:**

*IKZF1* silencing impairs the transcription of glucocorticoid-responsive genes; this effect is enhanced by concomitant loss of *BTG1*. These results demonstrate the molecular mechanism by which the combination of both genetic deletions might contribute to higher relapse rates in B-ALL.

## Introduction

B-cell precursor acute lymphoblastic leukemia (B-ALL) is the most common cancer diagnosed in children and is characterized by differentiation blockage and uncontrolled proliferation.^1^ Primary and secondary genetic events are essential for the establishment and progression of B-ALL.^2^ One recurring alteration damages the IKAROS protein, which normally orchestrates the lymphoid expression program through the recruitment of protein complexes that modify cellular epigenetic signatures.^3^ In pediatric B-ALL, *IKZF1* deletion occurs in 15% of patients and is associated with a higher risk of relapse, thus indicating its importance as a prognostic biomarker.^4^, ^5^, ^6^, ^7^

*BTG1*, another commonly affected gene in B-ALL, encodes a tumor suppressor protein. *BTG1* deletions are found in ∼10% of these cases and, although these genetic lesions alone are not associated with a worse disease outcome, they considerably increase the relapse risk when occurring in combination with *IKZF1* deletions.^8^ The interplay between these two genetic alterations on patient prognosis might be attributed to their influence on glucocorticoid response. Dexamethasone is one of the most recurrently used glucocorticoids to treat B-ALL, since it promotes apoptosis and inhibits pro-inflammatory cytokines.^9^, ^10^, ^11^ Therefore, glucocorticoids are essential components of B-ALL treatment, and a deficient glucocorticoid response might cause disease recurrence.^12^

A randomized clinical trial that enrolled patients newly diagnosed with B-ALL showed that dexamethasone-based therapy is correlated with a higher event-free survival and lower relapse rates, particularly of those involving the central nervous system.^13^ The cellular response to glucocorticoid relies on its corresponding receptor (NR3C1), which translocates into the nucleus upon ligand binding to act as a transcription factor. The NR3C1⎼glucocorticoid complex binds to specific DNA sequences (namely glucocorticoid response elements), thus resulting in transcriptional activation or repression of several target genes, such as *DUSP1* and *SGK1*, inducing apoptosis in blast cells.^14^ On the other hand, FBXW7 promotes NR3C1 ubiquitylation and proteasomal degradation.^15^^,^^16^ In view of the complexity involved in the human response to glucocorticoids and its pivotal importance for B-ALL treatment, it is necessary to understand the genetic features of leukemia that may disrupt this signaling pathway. This study evaluated whether *IKZF1* and/or *BTG1* silencing could impact the cell viability and transcript levels of glucocorticoid-responsive genes compared to dexamethasone treatment using the B-ALL 207 cell line as the experimental model.

## Material and Methods

### Cell culture

The 207 cell line (kindly provided by Dr. Martin Bonamino from INCA) is derived from a patient sample at the time of second relapse.^17^ The absence of recurrent gene fusions (*ETV6::RUNX1, TCF3::PBX1, BCR::ABL1, KMT2A::AFF1* and *KMT2A::ENL*) was verified in the 207 cell line by real time PCR (RT-PCR). In addition, no deletion of *IKZF1* or *BTG1* was identified by multiplex ligation-dependent probe amplification analysis. Cells were cultured in Roswell Park Memorial Institute (RPMI) 1640 medium supplemented with 10% fetal bovine serum (FBS) (Sigma-Aldrich), 2 mM L-glutamine (Sigma⎼Aldrich), 100 U/mL penicillin and 10 μg/mL streptomycin (Sigma-Aldrich), and kept with 5% CO_2_ at 37°C.

### Small interfering RNA design and transient transfection

Small interfering RNA (siRNA) sequences were designed using the Block-it^TM^ RNAi design platform (Invitrogen) (Supplementary Table S1). A total of 2 × 10^6^ cells were transfected with 100 nM of single or concomitant siRNA-specific sequences targeting *BTG1* and *IKZF1* (named si*BTG1* and si*IKZF1*, respectively) by electroporation using the 1SM buffer^18^ and Nucleofector 2B™ (Lonza). siRNAs scrambled sequences (siScramble) were used as controls. Gene silencing was evaluated at the 36 h timepoint and cells were kept in 2 mL of RPMI 1640 medium with 20% FBS and 5% carbon dioxide at 37°C (Supplementary Figure S1a–b). In order to guarantee that the silencing of both genes would remain during the pharmacological treatment, a period of 72 hours of silencing was also evaluated (Supplementary Figure S1c).

### Dexamethasone half-maximal inhibitory concentration (IC50) determination

The 3-[4,5-dimethylthiazol-2-yl]-2,5-diphenyltetrazolium bromide (MTT) colorimetric assay was used to determine the half-maximal inhibitory concentration (IC50) of the 207 cell line upon the use of dexamethasone. Cells were plated in 96-well plates containing 3 × 10^4^ cells in 100 µL of RPMI 1640 medium supplemented with 20% FBS. After 24  h, 100 µL of dexamethasone (0.25 mM to 5 mM) diluted in RPMI 1640 medium supplemented with 20% FBS was added. After 36 h, 10 µL of 0.5 mg/mL MTT solution (Sigma-Aldrich) was used to measure mitochondrial dehydrogenase activity by reading at 538 nm in a Spectramax 190 microplate reader (Molecular Device).

### Cytotoxicity assays

**Figure 1:**
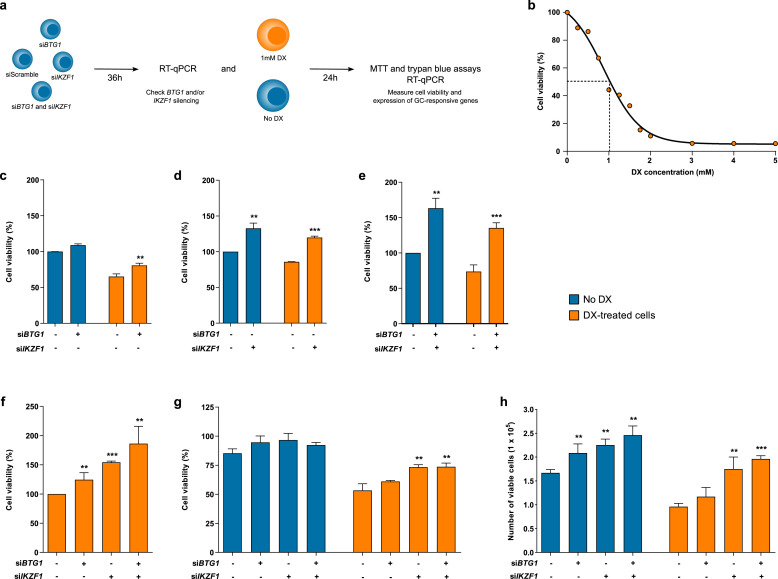
Cell viability of B-ALL cell line after isolated or combined silencing of *BTG1* and *IKZF1* in response to dexamethasone treatment. (a) Experimental design of this study, as detailed in the methodology. (b) The viability of 207 cells was assessed after 24 h of dexamethasone treatment at different concentrations. Subsequently, the proportions of viable cells silenced were measured using the MTT assay for (c) *BTG1*, (d) *IKZF1* and (e) both *BTG1* and *IKZF1* before and after treatment with dexamethasone. Also, the percentage of viable cells was measured using the MTT assay after 24 h of dexamethasone treatment and 36 hours of gene silencing (f). The results are compared between control cells (siScramble) and *BTG1*/*IKZF1* silenced cells. The cell viability was also measured as a percentage (g) and absolute count (h) of the cell number using the trypan blue exclusion assay. The results were obtained from three independent experiments, and statistical analyses were performed using the Student's t-test. *P-*values <0.05 were considered significant: *p-value *<*0.05; **p-value *<*0.005; ***p-value *<*0.001 (unpaired t-test).

Cell viability was evaluated in response to transfection of siRNAs and administration of 1 mM dexamethasone. The quantification of viable cells was based on MTT and trypan blue exclusion assays (Figure 1a). Cells were counted in a Neubauer chamber, where 10 uL of a solution with trypan Blue and cell suspension (1:1 dilution) was added. MTT assays of glucocorticoid-treated cells were performed using the same procedure.

### Gene expression

RNA extraction was performed using the Trizol™ reagent (Invitrogen), following the manufacturer's instructions. RNA integrity was observed by gel electrophoresis and quantification of nucleic acids using the NanoDrop™ device (ThermoScientific). RNA (1 µg) of was treated with Ambion DNAse I (RNAse free) and reverse transcribed into cDNA using random oligonucleotides and SuperScript™ II Reverse Transcriptase (Invitrogen). Then, cDNA synthesis and integrity were confirmed by amplification of the *GAPDH* transcript. Oligonucleotides for each target gene (*BTG1, BTG2, IKZF1, SGK1, DUSP1, FBXW7* and *NR3C1*) were self-designed (Supplementary Table S2), and *β-actin* (*ACTB*) was used as reference gene.^19^ Amplifications were performed using the GoTaq® quantitative PCR Master Mix (Promega) assay and the 7500 Real-Time PCR System™ (Applied Biosystems). Analyses were performed using the relative quantification method by ΔΔCq calculation.

### Statistical analysis

Differences between groups were compared using the Student's t-test. Statistical results and graphs were obtained using the GraphPad Prism 5 software (GraphPad™ Software, San Diego, CA, USA) with p-values <0.05 being interpreted as significant.

## Results

### Determination of inhibitory concentration of dexamethasone in 207 cells

To establish the ideal dexamethasone concentration and the time of drug exposure in our experiments, 207 cells were treated with increasing concentrations of dexamethasone (0⎼5 mM). Cell viability was evaluated by MTT assays after 24, 48, and 72 h. The cytotoxic effect of dexamethasone was similar, regardless of the time of drug exposure (Supplementary Figure S2). The IC50 after 24 hours of dexamethasone treatment was 1 mM (Figure 1b). Hence, this condition was chosen in the following experimental assays.

### The combined suppression of *BTG1* and *IKZF1* expression is associated with increased viability of dexamethasone-treated cells

MTT assays were performed to evaluate cell viability in response to silencing of *BTG1, IKZF1* or both transcripts in combination. *BTG1* silencing did not result in any significant alteration in cell viability in a dexamethasone-free condition. Conversely, dexamethasone-treated cells displayed reduced viability (Figure 1c), although si*BTG1*-transfected cells presented with 24% higher viability in comparison to siScramble-transfected cells (*p*-value = 0.0052). Of note, si*IKZF1* transfected cells had even higher viability, and isolated *IKZF1* (Figure 1d) or concomitant *BTG1* and *IKZF1* silencing (Figure 1e) using specific siRNAs resulted in 32% (*p*-value <0.0016) and 63% (*p*-value *<*0.0015) increases in cell viability, respectively, when compared to their respective siScramble untreated cells. Additionally, dexamethasone-treated cells transfected with si*IKZF1* (Figure 1d) or the combination of both si*BTG1* and *siIKZF1* (Figure 1e) had respective 40% (*p*-value *<*0.0001) and 84% (p-value *=* 0.008) higher cell viabilities compared to siScramble transfected cells.

The percentage of viable cells increases gradually after transfection of either si*BTG1*, si*IKZF1*, or si*BTG1* plus si*IKZF1* based on MTT data (Figure 1f)*. BTG1* silencing resulted in a 25% increase of cellular viability (p-value *=* 0.023), while *IKZF1* silencing displayed a more relevant role in the establishment of this phenotype, elevating cellularity by 54% (*p*-value <0.0001). The most relevant alteration was noticed after concomitant silencing of both *BTG1* and *IKZF1*, which raised the cell viability to 86% (*p*-value *=* 0.007). This result was validated by trypan blue exclusion counting analysis (Figure 1g⎼h). Although dexamethasone treatment reduced the proportion of viable cells, either isolated or combined transfection of si*IKZF1* and si*BTG1* resulted in higher cell viability compared to siScramble transfected cells (Figure 1g; *p*-value *=* 0.0049 and *p*-value *=* 0.006, respectively). The si*BTG1* transfected cells presented with a modest increase in cell viability, although not statistically significant (*p*-value *=* 0.08). Additionally, the number of viable cells prior to and after dexamethasone treatment was evaluated (Figure 1h). Although *BTG1* silencing did not impact cell viability after dexamethasone treatment (*p*-value *=* 0.1510), *IKZF1* silencing resulted in an increased number of viable cells before (*p-*value *=* 0.0021) and after (*p-*value *=* 0.0062) dexamethasone treatment. Similarly, simultaneous transfection of si*BTG1* and si*IKZF1* either prior to (*p-*value = 0.0026) or after (*p-*value *<*0.0001) dexamethasone treatment was associated with a greater number of viable cells. Nevertheless, no significant differences were observed in the number of viable cells between *IKZF1* knockdown and *BTG1* plus *IKZF1* silencing, before (*p-*value = 0.1947) or after (*p-*value = 0.2335) dexamethasone treatment.

### The transcriptional level of genes related to glucocorticoid-response was modified upon *BTG1* and *IKZF1* silencing

**Figure 2:**
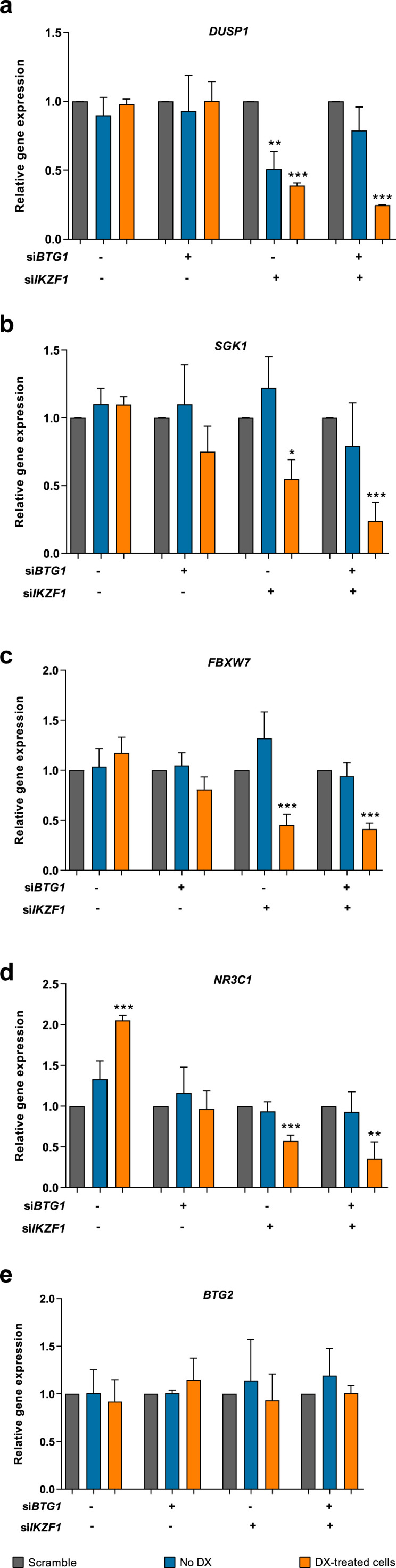
Transcript expression of glucocorticoid response genes following *BTG1* and/or *IKZF1* silencing and dexamethasone treatment. The 207 B-ALL cell line was treated with 100 nM of small interfering RNA (siRNA) for 36 h followed by treatment with 1 mM dexamethasone for 24 h. The expressions of (a) *DUSP1*, (b) *SGK1*, (c) *FBXW7*, (d) *NR3C1*, and (e) *BTG2* genes were evaluated for all silencing conditions. The relative gene expressions were calculated based on the ΔΔCT quantification method using the values obtained from three independent experiments. Statistical analyses were performed using the Student's t-test, and *p-*values <0.05 were considered significant: *p-value <0.05; **p-value <0.005; ***p-value *<*0.001 (unpaired t-test).

To determine whether *BTG1* and/or *IKZF1* silencing modulated the expression of genes related to glucocorticoid response, the transcript levels of *DUSP1, SGK1, FBXW7*, and *NR3C1* were measured (Figure 2a⎼d). *DUSP1* expression was not significantly modified in control or in si*BTG1* transfected cells either before or after dexamethasone treatment. On the other hand, *IKZF1* silencing resulted in 2-fold (*p-*value *=* 0.0028) and 2.6-fold (*p-*value *<*0.0001) reductions of *DUSP1* expression before and after dexamethasone treatment, respectively. However, there was no statistical difference in *DUSP1* transcript levels before and after dexamethasone treatment. Additionally, concomitant si*BTG1* and si*IKZF1* transfection resulted in a 4-fold lower *DUSP1* expression in response to dexamethasone treatment (*p-*value <0.0001) when compared to scrambled siRNA control (Figure 2a).

In agreement, *SGK1* expression was not altered in si*BTG1* or siScrambled cells. Yet, *IKZF1* silencing resulted in 1.8 lower *SGK1* expression upon dexamethasone treatment (*p-*value *=* 0.0056). The same finding was observed for concomitant silencing of *BTG1* and *IKZF1*, in which *SGK1* expression was reduced ∼4-fold (*p-*value *=* 0.0007) (Figure 2b).

Similarly, *BTG1* silencing did not affect *FBXW7* expression after dexamethasone treatment. Conversely, both isolated *IKZF1* silencing and combined *BTG1* plus *IKZF1* silencing resulted in significant reductions of *FBXW7* expression after dexamethasone treatment: 2.2-fold (*p-*value *=* 0.0010) and 2.4-fold (*p-*value *<*0.0001) reductions, respectively (Figure 2c). Of note, dexamethasone treatment increased the *NR3C1* transcript levels by 2-fold (*p-value <*0.0001). The transcript levels of *NR3C1* were not significantly altered in response to *BTG1* silencing. On the other hand, isolated *IKZF1* and the combination of *BTG1* and *IKZF1* silencing clearly indicated 1.7-fold (*p-*value *=* 0.0005) and 2.8-fold (*p-*value *=* 0.0055) reductions of the *NR3C1* transcript expression after dexamethasone treatment (Figure 2d). Nevertheless, the *NR3C1* levels between the single *IKZF1* and double silencing were similar (*p-*value *=* 0.1634).

### *BTG2* does not present a compensatory expression in *BTG1*-silenced cells

*BTG1* is a member of an anti-proliferative gene family that regulates cell growth and differentiation. Considering that *BTG2* is a paralog of this gene, its expression was measured to investigate whether it could compensate for *BTG1* silencing (Figure 2e). The data of this study indicate that *BTG2* expression does not undergo significant changes in any of the schemes evaluated, in which cells were transfected with siScramble (*p-*value *=* 0.5744), si*BTG1* (*p-*value *=* 0.3208), si*IKZF1* (*p-*value *=* 0.6931) or the combination of both si*BTG1* and si*IKZF1* (*p-*value *=* 0.8707). Therefore, *BTG2* does not compensate for the lack of *BTG1* expression in this B-ALL experimental model.

## Discussion

Despite advances in genomics and clonality studies which assessed the impact of secondary genetic events (e.g. *IKZF1* and *BTG1* deletions) on the establishment and progression of B-ALL,^20^^,^^21^ changes regarding the drugs used in medical practice were discrete over the last fifty years.^22^ In this perspective, glucocorticoids stand out among the main drugs used to treat this disease since they activate a gene transcription program that promotes apoptosis.^11^^,^^23^ It has been well documented in *ETV6::RUNX1*-positive patients that copy number alterations within genes related to glucocorticoid response are directly linked to treatment resistance and the occurrence of relapses.^24^ Due to the importance of glucocorticoid for B-ALL treatment, it is fundamental to understand its mechanisms of resistance. Here, this study aimed to investigate whether the disruption of *IKZF1* and *BTG1* synergistically promote glucocorticoid resistance.

First, although *BTG1* silencing resulted in higher cell viability when compared to its scramble, it did not result in significant expression alterations of glucocorticoid response genes, such as *DUSP1, SGK1, FBXW7* and *NR3C1*. A previous study used shRNA to silence *BTG1* in RS4;11 cells. After prednisolone and dexamethasone treatment, the authors observed an increase in cell viability due to *BTG1* silencing, as well as a reduction in the expression of genes related to glucocorticoid response.^25^ These partially discordant results regarding the expression levels of genes related to glucocorticoid response may be attributed to the different cell lines used in the two studies. Nonetheless, two other studies ⎼ one carried out with 116 patients with Down syndrome and ALL, and the other including 533 B-ALL patients ⎼ demonstrated that isolated *BTG1* deletion was not able to impact the prognosis of the patients.^8^^,^^26^ Taken together, these data suggest that *BTG1* deletions per se are not able to affect the response to glucocorticoid in a way that could modify the prognostic risk of patients.

Because no significant alterations in *DUSP1, SGK1, FBXW7* and *NR3C1* expressions after *BTG1* silencing were observed in this study, we hypothesized that BTG2 could compensate for the absence of BTG1. Although the BTG2 protein has a similar structure to BTG1, including the PRMT1 binding domain that is exclusive to these BTG/TOB family members,^27^ a compensatory expression was not observed in this experiment. Notably, there are relevant differences regarding the cellular pathways triggered by BTG1 and BTG2, including the fact that BTG2 response is p53 dependent.^28^ We speculate that BTG2 activity might have been compromised due to a *TP53* mutation observed in the 207 cell line,^29^ thus reducing its impact on the viability of *BTG1* silenced cells.

In contrast to *BTG1*, it is well described that *IKZF1* deletions confer a worse prognosis for patients with B-ALL.^4^^,^^30^ The association of *IKZF1* deletions with minimal residual disease (MRD) data predicts up to 79% of cases with disease relapse.^31^, ^32^, ^33^ Interestingly, a cohort study showed that concomitant *IKZF1* and *BTG1* deletions result in an even worse prognosis for these patients.^8^ The authors also evaluated the glucocorticoid responses in murine cells derived from crossings of mice with *Ikzf1* haplodeficiency and those subjected to btg1 silencing. Mice with btg1 silencing only presented a similar survival rate to the wild-type group. In contrast, mice with *Ikzf1* haplodeficiency had a worse survival, which was even poorer when occurring together with btg1 deficiency. In consonance, this study found that isolated *IKZF1* silencing and simultaneous *BTG1* and *IKZF1* silencing in dexamethasone-treated cells promoted a remarkable increase of their viability. This result corroborates with another study showing that cells isolated from *Ikzf1* haplodeficient mice are resistant to glucocorticoid treatment,^34^ and indicates the respective central and adjuvant roles of *IKZF1* and *BTG1* in promoting a resistant phenotype to dexamethasone.

In opposition to the results obtained with the *BTG1* silenced cells, either the isolated *IKZF1* or *BTG1* plus *IKZF1* silencing resulted in marked alterations of, not only cell viability, but also the expression of glucocorticoid response genes, thus reinforcing the relevance of the latter genes in the leukemic context. For instance, depending on the cellular context, *DUSP1* acts either as a tumor suppressor or an oncogene.^35^^,^^36^ DUSP1 can act as an anti-inflammatory effector through dephosphorylation of the mitogen-activated protein kinase (MAPK) in response to glucocorticoids. A study using *Dusp*^−/−^ mouse macrophages showed that the anti-inflammatory response produced by dexamethasone is partially impaired in this context.^37^ In accordance, *SGK1* expression was also reduced about 4-fold when both *IKZF1* and *BTG1* were silenced. Since *SGK1* has glucocorticoid-responsive elements, its transcription is induced early and remains active for hours.^38^^,^^39^ Therefore, we may infer that such reduced expressions of *SGK1* and *DUSP1* are directly related to a lower expression of the NR3C1 receptor in these *IKZF1* plus *BTG1* silenced cells, which reduces NR3C1 binding to those responsive-elements.

Furthermore, IKZF1 modulates AKT activity, which phosphorylates NR3C1 and inhibits its nuclear translocation.^40^^,^^41^ Notably, one study observed that glucocorticoid response failure in T-ALL was related to the presence of clones containing pre-existing or *de novo NR3C1* mutations.^42^ Therefore, a pan-PI3 kinase inhibitor may enhance the dexamethasone response based on the inhibition of AKT phosphorylation on NR3C1. Moreover, another study identified deletions in various actors of glucocorticoid signaling in 58% of relapsed B-ALL with the *ETV6::RUNX1* fusion. Among these copy number alterations, *BTG1* deletions were the most common in up to 35%. Despite the role of BTG1 in the regulation of the glucocorticoid pathway, only the *NR3C1* and *ETV6* deletions were correlated with poor MRD response. The authors also performed *in vitro* and xenograft mouse models to reconstitute wild-type NR3C1 expression in glucocorticoid-resistant mutant REH cells, and observed that abnormal NR3C1 is correlated with glucocorticoid resistance and a greater incidence of relapses among patients.^24^

The present study showed that *IKZF1* silencing resulted in a reduction of *NR3C1* expression, which was potentiated by concomitant *BTG1* silencing. A recent study also observed that the disruption of IKZF1 leads to reduced *NR3C1* expression.^41^ The loss of IKZF1 activity resulted in a prominent increase of dexamethasone resistance, which was partially diminished after the induction of IK1 transcript expression. Additionally, IKZF1 depletion accompanied the reduction of *NR3C1* expression and, as a consequence, a reduction of the expression of genes that promote glucocorticoid anti-inflammatory response. In this context, we assume that *IKZF1* silencing leads to an increased AKT activity and higher NR3C1 phosphorylation, which impairs appropriate response to the drug. Another player of the glucocorticoid response is FBXW7, which is responsible for targeting NR3C1 to the proteasome.^43^^,^^44^ The current study indicated that *IKZF1* silencing and concomitant *IKZF1* and *BTG1* silencing accompanied a reduction in *FBXW7* expression. Another study also observed the same finding in *IKZF1* silenced RS4;11 cells under glucocorticoid treatment.^34^ Considering that FBXW7 is responsible for ubiquitinating and directing the NR3C1 receptor for degradation in the proteasome, loss of function mutations within *FBXW7* would confer a better response to glucocorticoid.^16^ However, further studies are needed to fully understand this question.

We are aware this study had some limitations. First, experiments were performed with only one B-ALL cell line. Second, the expression levels of target genes (*IKZF1* and/or *BTG1*) in siRNA-transduced cells were validated at transcript, but not at protein level. Third, although functional screenings were performed at the timepoint of significant reduction of *IKZF1* and/or *BTG1* expression, we did not achieve a prolonged genetic knockdown, which may have limited the observed effects. Fourth, our experimental assays were restricted to dexamethasone treatment, and other glucocorticoids (such as prednisolone) were not tested. On the other hand, our findings allow a better understanding of the role of *BTG1* and *IKZF1* deletions in modulating glucocorticoid response, thus helping to unravel the resistance phenomenon in leukemia cells displaying this genotype (Supplementary Figure S3). The present study shows that loss of *IKZF1* expression is sufficient to induce glucocorticoid resistance, while loss of *BTG1* expression is a cooperative event which boosts this phenotype. Notably, data is provided in the defined context of a B-cell line model, a subgroup of B-ALL in which glucocorticoid resistance and disease relapse remain as significant clinical problems. Therefore, this study provides fundamental knowledge for the development of new therapeutic strategies to overcome treatment resistance and ensure a greater prognosis for patients.

## Conflicts of interest

The authors declare no competing interests.

## Appendix Supplementary materials

Image, application 1
