# Supplementary material for: IKZF1 and BTG1 silencing reduces glucocorticoid response in B-cell precursor acute leukemia cell line
Source: Hematol Transfus Cell Ther. 2024 Jul 26;46(Suppl 6):S163–70. doi: 10.1016/j.htct.2024.05.004 (PMC11726076; doi:10.1016/j.htct.2024.05.004)
Supplement: Supplementary file 1 [file mmc1.pdf]

Supplementary Material

IKZF1 and *BTG1* silencing reduces the glucocorticoid response in B-cell precursor acute leukemia cell line

Amanda de Albuquerque [a], Bruno A. Lopes [a,b], Renan Amphilophilo Fernandes [c], Etel Rodrigues Pereira Gimba [d,e], Mariana Emerenciano [a,b]

[a] Division of Clinical Research and Technological Development, Instituto Nacional de Câncer (INCA), Rio de Janeiro, RJ, Brazil;

[b] Group of Molecular Onco-hematology, Molecular Carcinogenesis Program, Instituto Nacional de Câncer (INCA), Rio de Janeiro, RJ, Brazil;

[c] Pharmacology and Medicinal Chemistry Program, Institute of Biological Sciences, Universidade Federal do Rio de Janeiro (UFRJ), Rio de Janeiro, RJ, Brazil;

[d] Department of Natural Sciences (RCN), Institute of Humanities and Health (IHS), Universidade Federal Fluminense (UFF), Rio de Janeiro, Brazil;

[e] Hematology-Molecular Oncology Program, Research Coordination, Instituto Nacional de Câncer (INCA), Rio de Janeiro, Brazil.

Table S1. Small interference RNA (siRNA) sequences.

Gene	siRNA	Sequence (5'-3')
Scramble	Sense	AGGUAGUGUAAUCGCCUUGTT
	Anti-sense	CAAGGCGAUUACACUACCUTT
IKZF1	Sense	GGGUCAAGACAUGUCCCAATT
	Anti-sense	UUGGGACAUGUCUUGACCCTT
BTG1	Sense	GGAUCGGGUUACCGUUGUATT
	Anti-sense	UACAACGGUAACCCGAUCCTT

Table S2. Primer sequences used for qPCR.

Gene	Orientation	Sequence (5'-3')
ACTB*	Forward	GGCGGCACCACCATGTACCCT
	Reverse	AGGGGCCGGACTCGTCATACT
IKZF1	Forward	GATGAAGAGAATGGGCGTTGC
	Reverse	CATTCATTTTCTCTCCCGAGGC
BTG1	Forward	GCTGCTGGCAGAACATTATAAACAT
	Reverse	CTCTCCAATTCTGTAGGACACTTCA
DUSP1	Forward	ATTTTGAGGGTCACTACCAGTACAA
	Reverse	TCCAGCATTCTTGATGGAGTCTATG
SGK1	Forward	TCTTTGAGCGCTAACGTCTTTCT
	Reverse	TCTGCTTCATGAAAGCGATGAGAAT
FBXW7	Forward	CCACCCAGAGGAAACTTTACAAAAA
	Reverse	CGCGGTACTCCTCTTTTCCTCTT
NR3C1	Forward	TTGGATTCTATGCATGAAGTGGTTG
	Reverse	AGCTAACATCTCGGGGAATTCAATA
BTG2	Forward	AGGCACTCACAGAGCACTAC
	Reverse	TGGGGTCCATCTTGTGGTTG

*Reference gene.

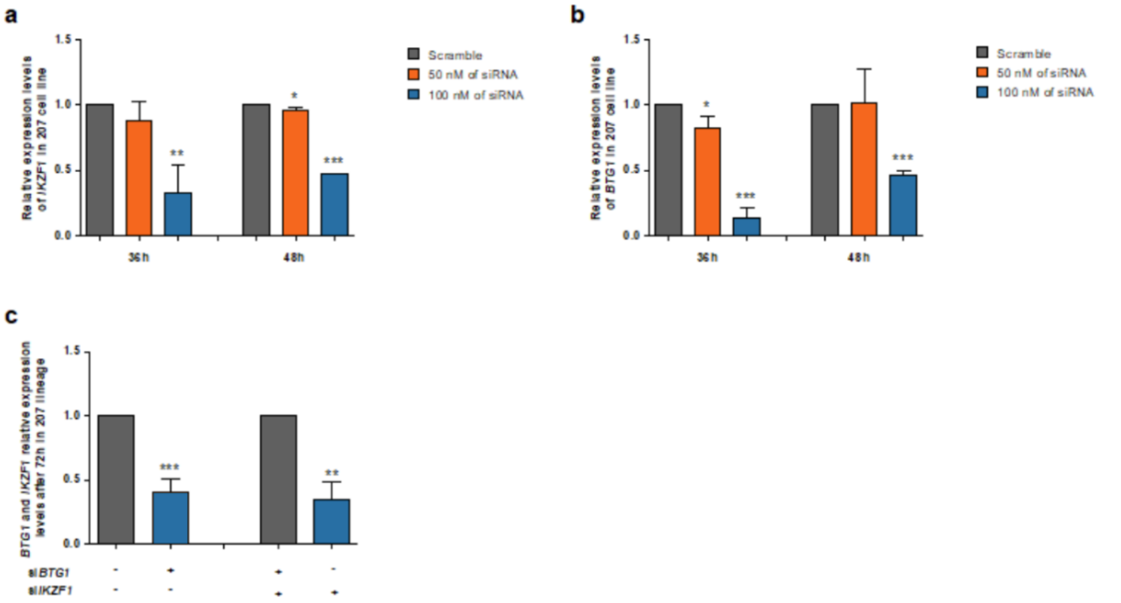

Supplementary Figure S1. Standardization of *BTG1* and *IKZF1* silencing and definition of optimal time and siRNA concentration for assay evaluation. Concentrations of 50 nM and 100 nM were used at times of 36 and 48 hours to observe the efficiency of concomitant inhibition of (a) *IKZF1* and (b) *BTG1*. After 36 hours of transfection using 100 nM siRNA resulted in 67% (P = 0.0051) of *IKZF1* and 87% (P < 0.0001) of *BTG1* silencing. Given the greater efficiency of the assay at a time of 36h and concentration of 100 nM siRNA for both genes, (c) the time of 72 hours of transfection was tested in order to assure that there would be inhibition throughout the pharmacological treatment with DX, resulting in 60% (P = 0.0006) and 65% (P = 0.001) of silencing to *IKZF1* and *BTG1*, respectively. Transcriptional expression of both genes was evaluated using RT-qPCR, where *ACTB* (encoding β-actin) was used as the reference gene. The ΔΔCT quantification method was used to calculate the relative expression, and statistical analyzes were performed using Student's t test. P values < 0.05 were considered significant: *P < 0.05; **P < 0.005; ***P < 0.001 (unpaired t-test).

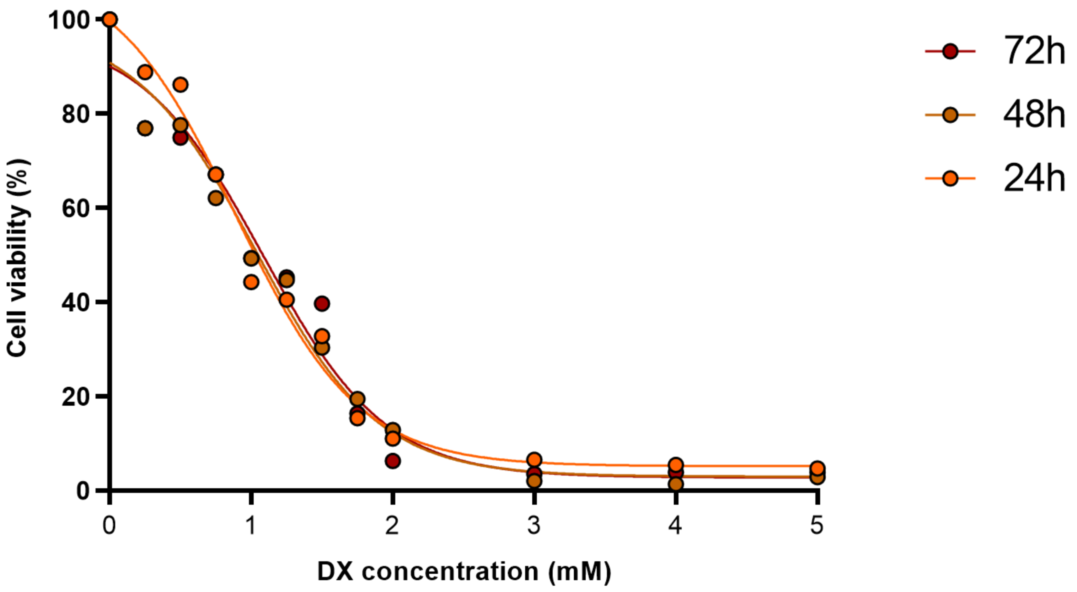

Supplementary Figure S2. Cell viability of the 207 cell line following DX treatment.

Cell viability was evaluated by MTT assay after 24 h, 48 h, and 72 h of DX treatment.

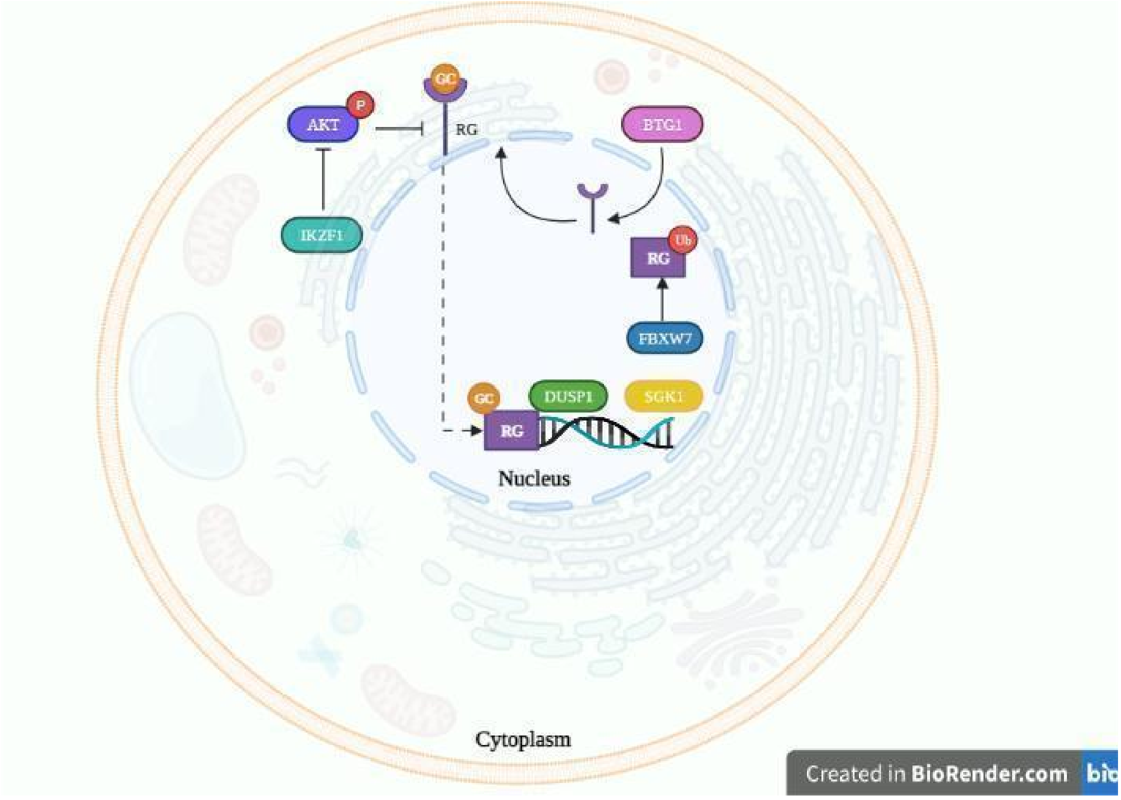

Supplementary Figure S3. Simplified illustration of the role of *BTG1* and *IKZF1* in the GC response pathway. IKZF1 regulates the phosphorylation status of the AKT protein, preventing it from phosphorylating the glucocorticoid receptor (GR). In turn, BTG1 is directly related to the amplification of this pathway by potentiating the self-induction of the receptor after induction by GC. The ligand-receptor binding allows its homodimerization and translocation into the nucleus. There it works as a transcription factor for GC response genes, such as *DUSP1* and *SGK1*. Finally, once the GR activity is over, it is marked for degradation through ubiquitination by the FBXW7 protein. Created with BioRender.com
